# Impact of growth curve and dietary energy-to-protein ratio on productive performance of broiler breeders

**DOI:** 10.1016/j.psj.2021.101131

**Published:** 2021-03-15

**Authors:** J. Heijmans, M. Duijster, W.J.J. Gerrits, B. Kemp, R.P. Kwakkel, H. van den Brand

**Affiliations:** ⁎ De Heus Animal Nutrition B.V., Ede, The Netherlands; † Animal Nutrition Group, Department of Animal Sciences, Wageningen University, Wageningen, The Netherlands; ‡ Adaptation Physiology Group, Department of Animal Sciences, Wageningen University, Wageningen, The Netherlands

**Keywords:** broiler breeder, feed strategy, sexual maturity, egg production, egg weight

## Abstract

The impact of growth curve (**GC**) and dietary energy-to-protein ratio on productive performance of broiler breeder females was investigated from 0 to 60 wk of age. One-day-old pullets (n = 1,536) were randomly allotted to 24 pens according to a 2 × 4 factorial arrangement, with 2 GC (standard growth curve = **SGC** or elevated growth curve = **EGC**, +15%) and 4 diets, differing in energy-to-protein ratio (96%, 100%, 104%, or 108% **AME_n_**). Feed allocation per treatment was adapted weekly based on the desired GC, meaning that breeders fed the different diets within each GC were fed according to a paired-gain strategy. Linear and quadratic contrasts for energy-to-protein ratio for each GC were evaluated. Elevated growth curve breeders had an earlier sexual maturity (∆ = 4.1 d) than SGC breeders. Egg weight was higher for EGC breeders (∆ = 2.3 g) than for SGC breeders over the whole laying phase (22–60 wk). No differences between EGC and SGC breeders were observed on settable egg production. An increase in dietary energy-to-protein, at a similar BW, led to a linear increase in age at sexual maturity (β = 0.14 d/% AME_n_). From 22 to 40 wk of age, an increase in dietary energy-to-protein ratio led to a linear decrease in egg weight (β = -0.06 g/% AME_n_), regardless of GC. An interaction between GC and dietary energy-to-protein ratio was observed on settable egg production in this phase. An increase in dietary energy-to-protein ratio led to a linear decrease on settable egg production, which was more profound in EGC breeders (β = -0.70 eggs/% AME_n_) than in SGC breeders (β = -0.19 eggs/% AME_n_). From 41 to 60 wk of age, an interaction between GC and dietary energy-to-protein ratio was observed on egg weight. In the EGC, an increase in dietary energy-to-protein ratio led to a linear decrease in egg weight (β = -0.13 g/% AME_n_), whereas in the SGC, a linear increase in egg weight was observed (β = 0.03 g/% AME_n_). From 41 to 60 wk of age, no differences between diets were observed on settable egg production. It can be concluded that a higher GC of breeders has beneficial effects on egg weight, while maintaining settable egg production. Feeding breeders a lower dietary energy-to-protein ratio stimulated productive performance of broiler breeder hens, mainly during the first phase of lay. This effect was more profound when breeders were fed according to a higher GC.

## INTRODUCTION

Broilers are genetically selected for high growth of muscle tissue and low body fat (Renema et al., 2007b; Zuidhof et al., 2014). This genetic selection has changed the body composition of broiler breeder hens as well (Eitan et al., 2014). Body fat mass in broiler breeder hens has decreased approximately 50% over the last 30 y (Eitan et al., 2014; Zuidhof, 2018). Several studies suggested that body fat mass of the broiler breeder hen plays an important role in sexual maturation (Bédécarrats et al., 2016; Zuidhof, 2018; Hadinia et al., 2020), egg production (Van der Klein et al., 2018), egg composition (Salas et al., 2017), and laying persistency (Van Emous et al., 2015). Concerns have recently been raised that a biological minimum of body fat mass may be approached or even reached in modern broiler breeder hens, which may endanger reproductive success (Van der Klein et al., 2018; Zuidhof, 2018; Hadinia et al., 2020).

In broiler breeders, changes in body composition are often due to either differences in growth curve or diet composition. A higher growth curve during rearing resulted in a higher body fat mass at 20 wk of age (Sun and Coon, 2005; Van Emous et al., 2013; Salas et al., 2019). Feeding a higher dietary energy-to-protein ratio during rearing, while maintaining a similar growth curve by pair-gaining, led to a higher body fat mass and a lower body lean mass at 20 wk of age compared to a lower dietary energy-to-protein ratio (Van Emous et al., 2013, 2015). In the indicated studies, however, contrasts in growth curve or diet composition were only maintained until 20 wk of age, resulting in breeders having the same body fat mass during production, irrespective of the initial BW and body fat mass differences at 20 wk of age (Sun and Coon, 2005; Van Emous et al., 2013, 2015; Salas et al., 2019). This may explain the absence of differences in egg production in these studies. Consequently, it can be hypothesized that differences in productive performance can only be reached when differences in growth curve or diet composition are maintained during the production phase.

This hypothesis is supported by Renema et al. (2001a) and Van der Klein et al. (2018), who maintained differences in growth curve during the production period and observed a higher total egg production when breeders were also heavier and fatter during production. Van der Klein et al. (2018) suggested that the higher productive performance of breeders on a higher growth curve was due to a higher body fat mass, but is it unclear whether or not a higher body lean mass of the heavier breeders might have played a role as well. Consequently, it remains unclear from these studies which of these variables are more dominant in determining effects on productive performance of broiler breeder hens.

Lesuisse et al. (2017, 2018) studied effects of a higher dietary energy-to-protein ratio, obtained by lowering the dietary crude protein content, during rearing and production. They observed a higher body fat mass during production when feeding a higher dietary energy-to-protein ratio, at a similar BW. In this study, a rather severe reduction of 25% in dietary crude protein led to a lower productive performance of the breeder hens. These findings suggest that a higher body fat mass, obtained by feeding less dietary crude protein, might not be beneficial for productive performance. It remains unclear whether a higher body fat mass, obtained by increasing dietary energy content, rather than a decreasing dietary crude protein content, might affect productive performance.

The aim in the current study was to disentangle effects of body composition and growth curve on productive performance, by feeding diets differing in energy-to-protein ratio at each of the two growth curves.

## MATERIALS AND METHODS

### Experimental Design

An experiment with female Ross 308 broiler breeders was set up as a 2 × 4 factorial arrangement, with 2 growth curves (**GC**) (standard growth curve = **SGC** or elevated growth curve = **EGC**) and 4 diets, differing in energy-to-protein ratio (defined as 96%, 100%, 104% or 108% AME_n_ diet). The experiment lasted from hatch to 60 wk of age. Feed allocation per treatment was adapted based the desired GC, meaning that breeders fed the different diets within each GC were fed according to a paired-gain strategy. All experimental protocols were approved by the Central Committee for Animal Experimentation (The Hague, the Netherlands), approval number 2018.W-0023.001.

### Breeders, Housing and Management

A total of 1,536 Ross 308 female broiler breeder day-old pullets were obtained from a 37 wk old grandparent flock (Aviagen-EPI, Roermond, The Netherlands). Pullets were randomly divided over 24 pens (64 pullets per pen), in a climate controlled room, in 3 blocks of 8 pens (n = 3 per treatment). Within each block, pens were randomly assigned to 1 of 8 treatments. Each pen had a floor area covered with wood shavings as bedding (1.75 × 2.80 m), an elevated floor (1.75 × 2.90 m) with plastic slats, and laying nests (1.75 × 0.60 m). Until 6 wk of age, the slats were covered with rearing paper (MS Schippers, Bladel, The Netherlands) and wood shavings. Until 20 wk of age, the laying nests were covered with plastic to prevent access or sight to the nest. Pullets and breeders were fed with a track feeding system (9 m feeding length), which was placed on the elevated floor, containing a grill to prevent roosters access to the feed (after 20 wk of age). During the first 2 wk of age, two additional feeding pans per pen were placed on the elevated floor in order to stimulate feed intake. Perches (7.2 m) were placed above the elevated floor. Water was available ad libitum via drinking nipples positioned above the elevated floor. Feed was provided once per day at 07.15 h from wk 0 to 21 of age and at 09.00 h from wk 21 to 60 of age. Room temperature was maintained at 36°C until 3 d of age. From 3 d of age onwards, temperature was gradually reduced to 20°C at 28 d of age and was maintained thereafter. Pullets were vaccinated according to a standard protocol (Poultry Vets, Diessen, The Netherlands) and reared at a photoperiod of 23L:1D (20 lux) at the day of arrival which gradually changed to 8L:16D (10 lux) at 21 d of age, which was maintained until 21 wk of age. Lights were on between 07.00 h and 15.00 h. At 21 wk of age, pullets were photo-stimulated by increasing the photoperiod instantly to 11L:13D (20 lux) and then gradually to 13L:11D (40 lux) at 23 wk of age. Lights were on between 03.00 h and 16.00 h.

At 20 wk of age, all pens were standardized to 45 breeders per pen closest to the average pen weight. At that age, 4 Ross 308 roosters of the same age were introduced per pen. Roosters were fed with one rooster feeding pan, which was placed above the littered area at a minimum of 50 cm height to prevent breeder access. Roosters were fed a commercial available rooster diet (2,725 kcal of AME_n_/kg, 134 g of CP/kg, 5 g digestible lysine/kg). Body weight, body condition, and mating activity of roosters were assessed every other week according to breeder recommendations (Aviagen, 2018). Inactive roosters were instantly replaced by sexually mature spike roosters.

### Experimental Diets and Feed Allocation

Pullets and breeders were fed according to a 5-phase feeding system. A starter 1 diet was fed from 0 to 3 wk, a starter 2 diet from 3 to 6 wk, a grower diet from 6 to 16 wk, a pre-breeder diet from 16 to 23 wk, a breeder 1 diet from 23 to 40 wk, and a breeder 2 diet from 40 to 60 wk of age. All diets were fed as mash. Feed was provided *ad libitum* from day of arrival until 2 wk of age. Thereafter, daily feed allocation was adjusted weekly to obtain 2 different GC. The SGC was according to the breeder recommendation (Aviagen, 2016a), whereas the EGC targeted to have a 15% higher BW throughout rearing and production. Within each GC, daily feed allocation was adjusted in the 96%, 104%, and 108% AME_n_ diets to obtain pair-gaining to the 100% AME_n_ diet. Within each phase and GC, all diets were formulated isonitrogenous. Dietary AME_n_ levels were increased linearly from 96% to 108% in 4 steps (96%, 100%, 104%, and 108%) relative to the standard (100%; Aviagen, 2016b). The higher AME_n_ levels were reached by exchanging cellulose and finely ground oat hulls for soy oil, lard, and maize starch. The ratio between crude fat and starch was kept similar in all diets within each feeding phase. First, the 96% and 108% AME_n_ diets were produced. The intermediate diets (100% and 104% AME_n_) were produced by homogeneous mixing 96% and 108% AME_n_ diets in a 2:1 (100% AME_n_) or 1:2 (104% AME_n_) ratio. Diets were analyzed on CP (NEN-EN-ISO 16634-1), crude fat (NEN-EN-ISO 6492-1999), and starch (NEN-ISO 6493) content. Ingredient composition with calculated and analyzed nutrient contents of the experimental diets is presented in Table 1.

**Table 1:** Dietary ingredients, and calculated and analyzed nutrients of diets (g/kg, as-fed basis).

Item	Starter 1 (0–21 d)		Starter 2 (22–42 d)		Grower (43–112 d)		Pre-breeder (113–160 d)		Breeder 1 (161–280 d)		Breeder 2 (281–420 d)	
Ingredient	96% AME_n_	108% AME_n_	96% AME_n_	108% AME_n_	96% AME_n_	108% AME_n_	96% AME_n_	108% AME_n_	96% AME_n_	108% AME_n_	96% AME_n_	108% AME_n_
Maize	450.0	450.0	500.0	500.0	400.0	400.0	500.0	500.0	440.0	440.0	460.0	460.0
Wheat	100.0	100.0	100.0	100.0	100.0	100.0	100.0	100.0	100.0	100.0	100.0	100.0
Soybean meal	240.9	245.1	141.3	146.3	76.1	80.7	48.9	52.8	149.8	152.5	130.5	133.4
Sunflower meal	50.0	50.0	90.0	90.0	150.0	150.0	165.0	165.0	80.0	80.0	90.0	90.0
Wheat middlings	-	-	-	-	100.0	100.0	25.0	25.0	-	-	-	-
Oat hulls (fine)	50.0	1.0	56.0	5.1	65.0	19.3	50.0	1.0	48.0	1.0	46.6	1.0
Cellulose	44.1	1.0	47.9	5.0	50.0	5.0	46.8	1.0	44.5	1.0	45.2	1.0
Soya oil	11.1	17.8	9.5	14.3	8.0	12.0	5.0	7.0	4.8	10.8	11.9	14.9
Lard	3.0	4.2	4.2	6.8	3.3	6.7	5.0	10.2	29.5	34.9	23.5	32.1
Maize starch	14.0	94.5	14.3	96.2	19.9	99.2	11.7	96.1	14.7	91r.6	1.0	76.9
Chalk	13.9	14.1	13.8	13.9	13.3	13.4	-	-	-	-	-	-
Limestone (coarse)	-	-	-	-	-	-	24.5	24.6	71.0	71.1	73.4	73.5
Monocalcium phosphate	9.8	9.2	10.5	9.9	5.4	4.9	5.8	5.2	6.0	5.5	6.5	5.9
Sodium bicarbonate	3.3	3.3	3.3	3.3	2.5	2.5	3.3	3.3	2.7	2.7	3.0	2.9
Salt	1.8	1.8	1.7	1.7	2.2	2.2	1.5	1.5	2.1	2.1	2.0	2.0
L-Lysine	1.73	1.69	1.88	1.80	0.23	0.15	1.63	1.58	0.44	0.42	0.36	0.34
L-Threonine	0.68	0.68	0.54	0.54	-	-	0.49	0.48	0.57	0.58	0.54	0.55
DL-Methionine	2.34	2.34	1.71	1.71	0.65	0.65	1.13	1.13	1.73	1.77	1.59	1.62
Choline Chloride-50%	0.8	0.8	0.8	0.8	0.8	0.8	1.5	1.4	1.4	1.3	1.5	1.4
Xylanase	0.1	0.1	0.1	0.1	0.1	0.1	0.1	0.1	0.1	0.1	0.1	0.1
Phytase	0.05	0.05	0.05	0.05	0.05	0.05	0.05	0.05	0.05	0.05	0.05	0.05
Premix rearing^1^	2.5	2.5	2.5	2.5	2.5	2.5	-	-	-	-	-	-
Premix laying^2^	-	-	-	-	-	-	2.5	2.5	2.5	2.5	2.5	2.5
Calculated content^3^												
AME_n_ (kcal/kg)	2,570	2,890	2,570	2,890	2,545	2,865	2,640	2,970	2,735	3,080	2,735	3,080
Crude protein	175.1	175.0	143.7	143.6	136.5	136.5	123.0	122.5	138.5	137.7	135.2	134.3
Crude fat	41.5	49.0	42.0	49.0	40.0	47.0	38.8	45.7	60.0	71.1	61.6	72.8
Crude fiber	77.1	37.7	88.0	48.3	111.5	71.5	105.6	64.3	81.4	42.0	85.2	43.9
Starch	379.5	446.9	408.6	477.5	371.5	438.5	407.5	480.4	368.2	434.4	373.8	436.0
Starch:fat	9.1	9.1	9.7	9.7	9.3	9.3	10.5	10.5	6.1	6.1	6.1	6.0
Linoleic acid	18.0	21.0	18.0	20.3	17.0	19.0	16.3	17.4	16.8	20.0	20.0	22.0
Digestible lysine	9.0	9.0	7.0	7.0	4.8	4.8	5.1	5.1	5.9	5.9	5.5	5.5
Calcium	9.8	9.8	9.8	9.8	8.9	8.9	13.1	13.1	31.0	31.0	31.0	31.0
Retainable phosphorus	4.1	4.1	4.1	4.1	3.3	3.3	3.2	3.2	3.2	3.2	3.2	3.2
Analyzed content												
Crude protein^4^	170.2	172.9	145.1	148.0	133.0	135.1	129.6	127.4	145.2	142.2	139.9	135.1
Crude fat^4^	37.0	43.2	38.3	44.3	39.0	42.4	33.1	41.1	57.6	66.8	58.2	67.3
Starch	401.0	463.0	408.0	472.0	377.0	431.0	415.6	486.3	376.4	436.8	371.7	432.5

1 Provided per kg diet: Vitamin A 10,000 IU; Vitamin D_3_ 3000 IU; Vitamin E 100 IU; Vitamin K 3.0 mg; Vitamin B_1_ 3.0 mg; Vitamin B_2_ 6.0 mg; Vitamin B_6_ 4.0 mg; Vitamin B_12_ 20 μg; Niacinamide 35 mg; D-pantothenic acid 15 mg; Folic acid 1.5 mg; Biotin 0.20 mg; Iron 40 mg; Copper 16 mg; Manganese 120 mg; Zinc 90 mg; Iodine 1.25 mg; Selenium 0.3 mg.

2 Provided per kg diet: Vitamin A 10,000 IU; Vitamin D_3_ 3000 IU; Vitamin E 100 IU; Vitamin K 5.0 mg; Vitamin B_1_ 3.0 mg; Vitamin B_2_ 12.0 mg; Vitamin B_6_ 5.0 mg; Vitamin B_12_ 40 μg; Niacinamide 55 mg; D-pantothenic acid 15 mg; Folic acid 2.0 mg; Biotin 0.40 mg; Iron 50 mg; Copper 10 mg; Manganese 120 mg; Zinc 90 mg; Iodine 2.0 mg; Selenium 0.3 mg.

3 Calculated according to CVB (2012).

4 Analyzed values were within boundaries of the analytical error.

### Observations and Measurements

Body Weight, Feed Allocation and Mortality***.*** Body weight was determined weekly before feeding by weighing a minimum of 20 (rearing phase) or 15 (production phase) randomly selected breeders per pen. Once every 3 (rearing phase) or 4 (production phase) wk, all breeders within a pen were weighed individually. At these moments, BW uniformity (SD and CV) was calculated for each pen. Feed allocation per pen (expressed as g/breeder per d) was recorded weekly and adjusted to reach a targeted BW gain among diets within each GC. Average daily feed allocation was calculated per pen per phase (from now on defined as rearing phase, first phase of lay, and second phase of lay; 0–21, 22–40, and 41–60 wk of age, respectively). Average daily nutrient intake per pen per phase was calculated by multiplying average feed allocation per pen per phase with the calculated nutrient content of the diet. Relative nutrient intake per phase was calculated also per pen and expressed as a percentage to the 100% AME_n_ within GC. Mortality was recorded daily per pen and included culled breeders. Mortality during the first 2 wk of age was excluded from analysis.

Abdominal Fat Pad. At 12, 16, 21, 24, 28, 31, 36, 46 and 60 wk of age, 2 breeders per pen were selected before feeding within ± 2.5% of the average BW of both GC. Breeders were euthanized by a percussive blow on the head, followed by cervical dislocation. Breeders were defeathered, dissected and the abdominal fat pad, including fat surrounding the gizzard and proventriculus, was weighed. Abdominal fat pad percentage was calculated as a percentage of live BW.

Egg Production Traits***.*** Eggs were collected daily per pen. Eggs were graded as settable or unsettable (small (<50 g), double yolked, abnormal shell, dirty, cracked, or floor eggs). Total egg mass of all settable, unsettable, and double yolked eggs was recorded daily per pen. Average egg weight of all eggs, excluding double yolked eggs, was calculated per pen per phase. Total number of eggs, settable eggs, and unsettable eggs was calculated per pen per phase (22–40 wk, 41–60 wk, and 22–60 wk). Age at sexual maturity (**ASM**) was defined as age at 50% production and was determined per pen by a linear interpolation of age in days at which breeders passed 50% rate of lay. Age at first settable egg was defined as age at 50 g egg weight and was determined per pen by a linear interpolation of age in days at which breeders passed 50 g egg weight. Peak egg production per pen was determined as a 3-wk moving average.

Feather Development. Feather cover score of 10 randomly selected breeders per pen was recorded at a 5-wk (rearing phase) or 10-wk (production phase) interval, starting at 5 wk of age. Feather cover was scored according to the method described by Bilcik and Keeling (1999). Scores, varying from 0 (intact feathers) to 5 (completely denuded area), were given to 4 body parts (back, wings, tail, and thighs). The average score of the 10 breeders per pen was calculated per body part. The average of 4 body parts was calculated as an average feather cover score. Feather weight, as a percentage of live BW, was determined at the same ages, of the same breeders, as abdominal fat pad weight was determined and additionally at wk 6 of age. Feather weight was calculated as the difference between live BW and defeathered BW.

### Statistical Analysis

Data on BW (plus SD and CV) and abdominal fat pad percentage were analyzed per measuring moment, due to heterogeneous variation between ages. Data on all other variables (feed allocation, nutrient intake, laying performance, feather development and mortality) were analyzed per phase or overall. All data were analyzed using the Restricted Maximum Likelihood variance component analysis procedure within a linear mixed model (Genstat 19th Edition, 2019). The model used was:

$$Y_{ijk}=\;\mu +GC_{i}+Diet_{j}+GC_{i}\;x\;Diet_{j}+Block_{k}+e_{ijk,}$$

Where *Y_ijk_* = the dependent variable, µ was the overall mean, *GC_i_* = the growth curve (i = SGC or EGC), Diet_j_ = the energy-to-protein ratio in the diet (j = 96%, 100%, 104%, or 108% AME_n_), *GC_i_* x *Diet_j_* = the interaction between GC and diet, *Block_k_* = block within the room (k = 1, 2 or 3), and *e_ijk_* = the residual error. Additionally, effects of dietary energy-to-protein ratio were analyzed as linear or quadratic contrasts, also within GC. Feather parameters were analyzed with the same model added with breeder age and interactions of the other factors with breeder age. Pen was used as experimental unit for all analyses. Distributions of means and model residuals were checked on homogeneity and normality. Not-normal distributed data (feather scores) were square root transformed before analyses. Least square differences were compared, using Fisher adjustments for multiple comparisons. Data are presented as LS means ± SEM. For transformed data, LS means of original data are presented, combined with *P*-values of the transformed data. All statements of significance are based on testing at *P* ≤ 0.05. Comparisons between treatments, presented in the tables, are based on the factorial analysis. The slope (β) of linear effects of dietary energy-to-protein ratio, also within GC, is presented in the results section.

## RESULTS

### Feed Allocation and Nutrient Intake

Daily feed allocations within a treatment were the same for all pens. In all phases feed allocation was on average higher for EGC breeders than for SGC breeders (*P* < 0.001; Table 2). To achieve pair-gaining, feed allocation in all phases decreased with an increasing energy-to-protein ratio. However, this decrease was not the same for both GC (GC x diet (linear) *P ≤* 0.001; Table 2).

**Table 2:** Average feed allocation (FA) and nutrient intake during rearing (0–21 wk), first phase of lay (22–40 wk), and second phase of lay (41–60 wk) of broiler breeders with 2 different growth curves (SGC = standard growth curve or EGC = elevated growth curve (+15%)) and 4 diets, differing in energy-to-protein ratio (96, 100, 104, or 108% AME_n_), fed from 0 to 60 wk of age.

		0–21 wk			22–40 wk			41–60 wk		
Item		FA (g/d)	Energy (kcal/d)	CP^1^ (g/d)	FA (g/d)	Energy (kcal/d)	CP^1^ (g/d)	FA (g/d)	Energy (kcal/d)	CP^1^ (g/d)
Growth curve (n = 12)										
SGC		66.6	182.2^b^	9.0	142.4	412.0	19.5	147.1	426.6	19.8
EGC		77.5	212.0^a^	10.5	169.9	491.3	23.3	162.7	471.6	21.9
SEM		0.1	0.1	0.1	0.3	0.8	0.0	0.19	0.51	0.03
Diet (n = 6)										
96% AME_n_		75.7	195.3^d^	10.2	168.4	459.3	23.1	169.1	462.6	22.9
100% AME_n_		73.2	196.6^c^	9.9	157.8	448.5	21.6	156.4	445.8	21.1
104% AME_n_		70.8	197.8^b^	9.6	151.5	447.9	20.7	150.4	445.8	20.2
108% AME_n_		68.4	198.5^a^	9.2	146.9	451.0	20.0	143.6	442.3	19.3
SEM		0.1	0.2	0.1	0.4	1.2	0.1	0.27	0.75	0.04
Treatment (n = 3)										
SGC	96% AME_n_	70.0^e^	180.5	9.5^e^	151.7^e^	413.7^c^	20.8^e^	158.9^c^	434.6^d^	21.5^c^
	100% AME_n_	67.6^f^	181.7	9.1^f^	144.4^f^	410.3^c^	19.8^f^	149.0^f^	424.6^ef^	20.1^f^
	104% AME_n_	65.4^g^	182.9	8.9^g^	138.7^g^	410.2^c^	19.0^g^	143.6^g^	425.8^e^	19.3^g^
	108% AME_n_	63.2^h^	183.5	8.5^h^	134.8^h^	413.9^c^	18.4^h^	136.8^h^	421.3^f^	18.4^h^
EGC	96% AME_n_	81.5^a^	210.1	11.0^a^	185.1^a^	505.0^a^	25.4^a^	179.3^a^	490.5^a^	24.3^a^
	100% AME_n_	78.7^b^	211.6	10.6^b^	171.2^b^	486.6^b^	23.5^b^	163.8^b^	467.0^b^	22.1^b^
	104% AME_n_	76.1^c^	212.7	10.3^c^	164.3^c^	485.7^b^	22.5^c^	157.1^d^	465.8^bc^	21.2^d^
	108% AME_n_	73.6^d^	213.5	9.9^d^	158.9^d^	488.1^b^	21.7^d^	150.4^e^	463.3^c^	20.2^e^
	SEM	0.1	0.2	0.1	0.7	1.8	0.1	0.1	1.1	0.1
*P*-value										
Growth curve (GC)		<0.001	<0.001	<0.001	<0.001	<0.001	<0.001	<0.001	<0.001	<0.001
Diet (factorial)		<0.001	<0.001	<0.001	<0.001	<0.001	<0.001	<0.001	<0.001	<0.001
Diet (linear)		<0.001	<0.001	<0.001	<0.001	0.001	<0.001	<0.001	<0.001	<0.001
Diet (quadratic)		0.17	0.08	0.28	<0.001	<0.001	<0.001	<0.001	<0.001	<0.001
GC x Diet (factorial)		<0.001	0.87	<0.001	0.004	0.003	0.003	<0.001	<0.001	<0.001
GC x Diet (linear)		<0.001	0.43	<0.001	0.001	0.001	0.001	<0.001	0.002	<0.001
GC x Diet (quadratic)		0.85	0.96	0.88	0.06	0.06	0.06	0.01	0.02	0.01

^a-h^LSmeans within a column and factor lacking a common superscript differ (*P* ≤ 0.05).

1 Based on the calculated CP content.

To achieve pair-gaining within SGC feed allocation of the different diets was adjusted with -0.6 g/% AME_n_, -1.4 g/% AME_n_, and -1.8 g/% AME_n_ for the rearing phase, first phase of lay, and second phase of lay, respectively. To achieve pair-gaining within EGC feed allocation of the different diets was adjusted with -0.7 g/% AME_n_, -2.1 g/% AME_n_, and -2.3 g/% AME_n_ for the rearing phase, first phase of lay, and second phase of lay, respectively (GC x diet (linear) *P ≤* 0.001; Table 2). On average daily feed allocation in EGC breeders was 10.9 g, 27.5 g, and 15.6 g higher than in SGC breeders for the rearing phase, first phase of lay, and second phase of lay, respectively (*P* < 0.001; Table 2).

Interactions between GC and diet (linear) were also observed for daily energy and CP intake in all phases (*P ≤* 0.002), with exception of daily energy intake during the rearing phase (*P* = 0.43). During the rearing phase, energy intake increased with 0.3 kcal/d/% AME_n_ (*P* < 0.001), in both GC. In the first phase of lay, energy intake of did not differ between the diets within SGC breeders, whereas energy intake decreased with -1.3 kcal/d/% AME_n_ (*P* = 0.001) within EGC breeders. In the second phase of lay, energy intake decreased in both GC with increasing dietary energy-to-protein intake, but this effect was more profound within EGC breeders (β = -2.1 kcal/d/% AME_n_) than within SGC breeders (β = -1.0 kcal/d/% AME_n_; *P* = 0.002). Within the SGC, daily CP intake decreased with -0.1 g/% AME_n_, -0.2 g/% AME_n_, and -0.3 g/% AME_n_ for the rearing phase, first phase of lay, and second phase of lay, respectively. Within the EGC, daily CP intake decreased with -0.1 g/% AME_n_, -0.3 g/% AME_n_, and -0.3 g/% AME_n_ for the rearing phase, first phase of lay, and second phase of lay, respectively (GC x diet (linear) *P ≤* 0.001; Table 2).

**Figure 1:**
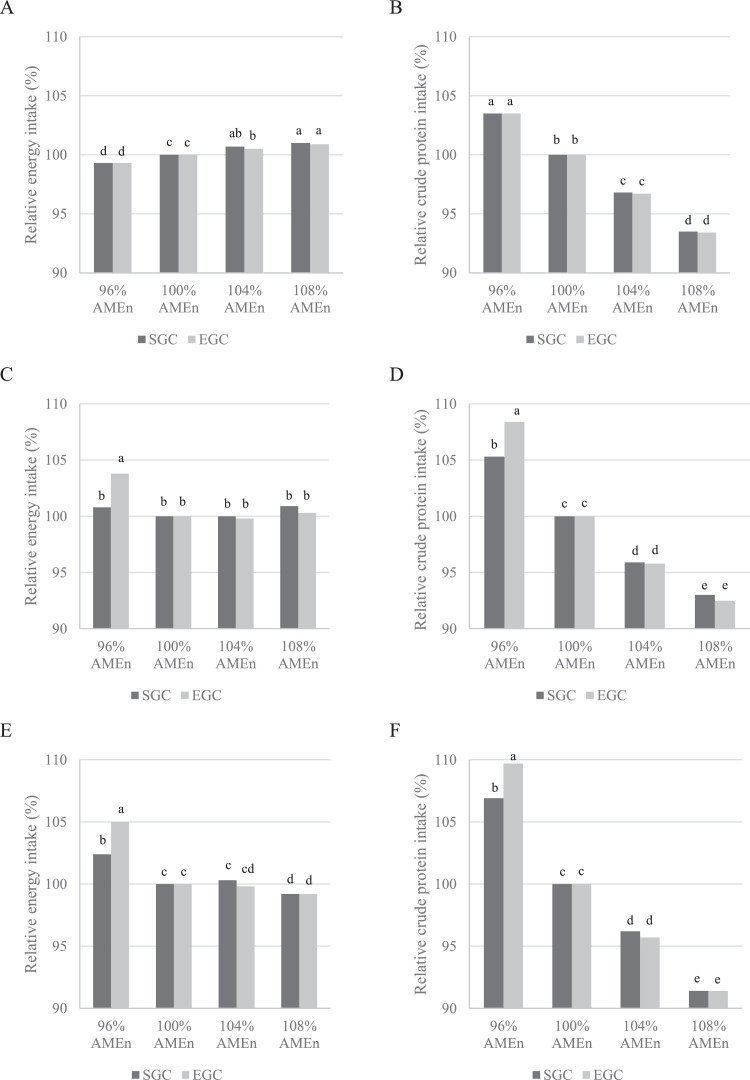
Energy and CP^1^ intake during the rearing phase (0–21 wk; A, B), first phase of lay (22–40 wk; C, D), and second phase of lay (41–60 wk; E, F), expressed as percentage relative to the 100% AME_n_ diet within growth curve, of broiler breeders with 2 different growth curves (SGC = standard growth curve or EGC = elevated growth curve (+15%)) and 4 diets, differing in energy-to-protein ratio (96, 100, 104, or 108% AME_n_), fed from 0 to 60 wk of age. ^a-e^LSmeans lacking a common superscript differ (*P* ≤ 0.05). ^1^Based on the calculated CP content.

Although significant differences were observed in energy intake, differences were rather small when expressed as relative difference to the 100% AME_n_ diet (Figure 1). Relative differences in CP intake, expressed as percentage to the 100% AME_n_ diet, were much larger (Figure 1). To achieve pair-gaining, relative differences in energy and CP intake did not significantly differ between GC for each diet in the rearing phase (Figure 1A and 1B). This means that the correction (percentage compared to the 100% AME_n_ diet) needed for pair-gaining in feed allocation per diet was similar for each GC. During the first and second laying phase, relative differences in energy and CP intake were similar for breeders in each GC on the 100%, 104% and 108% AME_n_ diets (Figure 1C–F). Again, this means the correction needed for pair-gaining in feed allocation was similar for each GC for these diets. However, this was not the case for the 96% AME_n_ diet. EGC breeders fed the 96% AME_n_ diet required a higher feed and thus nutrient intake for pair-gaining than SGC breeders fed the 96% AME_n_ diet during the laying phase (Figure 1C–F).

### Body Weight, Uniformity and Mortality

**Figure 2:**
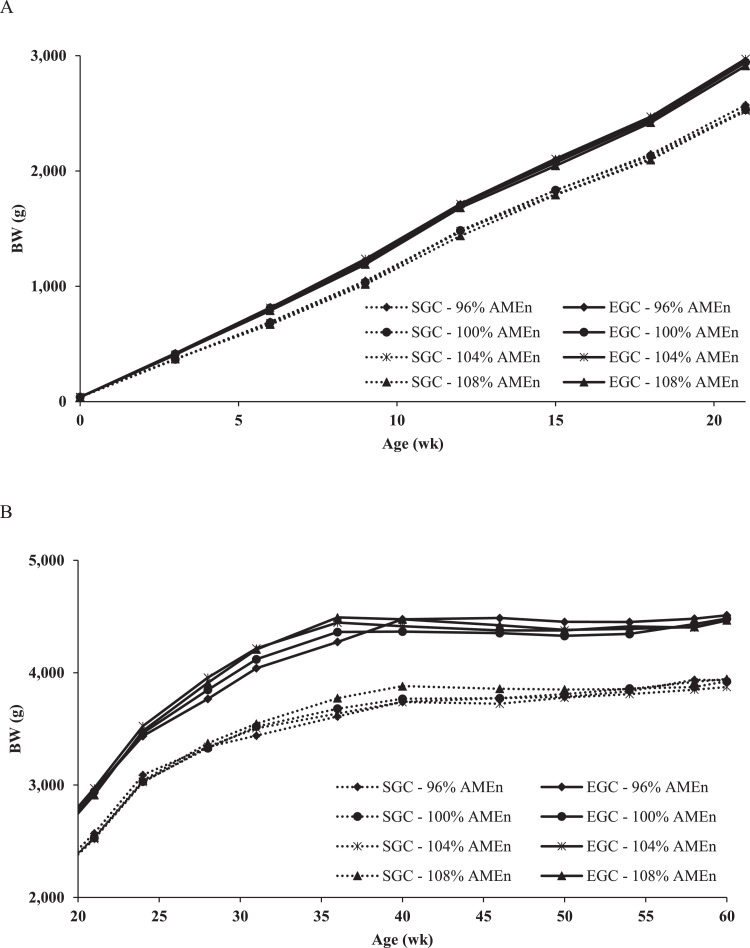
Body weight during the rearing phase (A; 0–21 wk) and production phase (B; 21–60 wk) of broiler breeders with 2 different growth curves (SGC = standard growth curve or EGC = elevated growth curve (+15%)) and 4 diets, differing in energy-to-protein ratio (96, 100, 104, or 108% AME_n_), fed from 0 to 60 wk of age.

Breeders on the different diets closely followed their targeted GC (SGC or EGC; Figure 2). Although daily feed allocations were adjusted weekly for each diet to obtain pair-gaining within each GC, temporary differences in BW among diets occurred. After adjustment in daily feed allocation, differences in BW disappeared.

**Figure 3:**
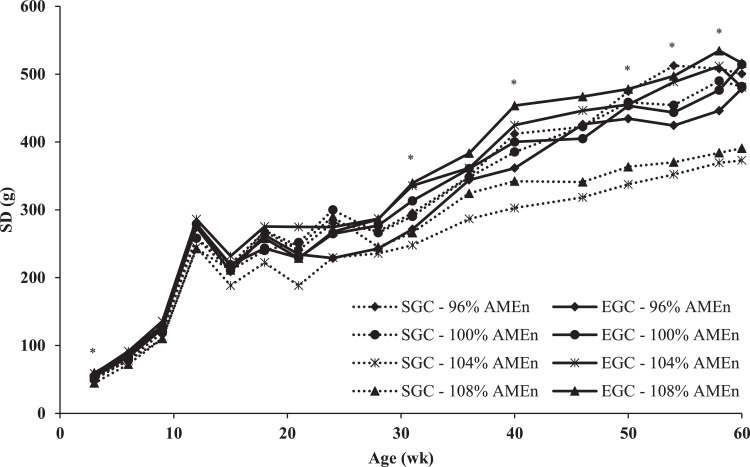
Uniformity, expressed as standard deviation (SD), of broiler breeders from 3 to 60 wk of age, with 2 different growth curves (SGC = standard growth curve or EGC = elevated growth curve (+15%)) and 4 diets, differing in energy-to-protein ratio (96, 100, 104, or 108% AME_n_), fed from 0 to 60 wk of age. *LSmeans within age with asterisk show a significant linear interaction effect of energy-to-protein ratio within GC on SD (*P* ≤ 0.05).

SD in BW was higher in the EGC than in the SGC up to 12 wk of age (*P* < 0.02; Figure 3), whereas the CV did not differ between GC (data not presented). BW uniformity (SD and CV) showed an interaction between GC and diet (linear) at 31, 40, and from 50 to 58 wk of age (*P* < 0.05). Within the EGC, a linear increase in dietary energy-to-protein ratio led to a linear increase in SD and CV (β = 6.1 g SD/% AME_n_ and β = 0.14 % CV/% AME_n_ on average, respectively). Within the SGC, a linear increase in dietary energy-to-protein ratio, led to a linear decrease in SD and CV (β = -9.5 g SD/% AME_n_ and β = -0.26 % CV/% AME_n_ on average, respectively).

The average mortality from 2 to 60 wk of age was 8.4%. No differences were observed in mortality between treatments (data not presented).

### Abdominal Fat Pad

No interaction between GC and dietary energy-to-protein ratio on abdominal fat pad percentage was observed (Table 3). From wk 16 until wk 46, with exception at 24 wk of age, EGC breeders had a higher abdominal fat pad percentage (∆ = 0.77 % on average; *P* < 0.05) than SGC breeders (Table 3). At 12, 24, and 60 wk of age, no differences in abdominal fat pad percentage were observed between GC. At 12 wk of age and from 21 to 36 wk of age, a linear increase in dietary energy-to-protein ratio led to a linear increase in abdominal fat pad percentage (β = 0.06 %/% AME_n_ on average; *P* < 0.05; Table 3). At wk 16, 46, and 60, no significant differences in abdominal fat pad percentage between diets were observed. Between 36 and 46 wk of age, abdominal fat pad percentage decreased for the 100%, 104%, and 108% AME_n_ breeders (∆ = -0.36 % on average), whereas the 96% AME_n_ breeders, showed an increase in abdominal fat pad percentage (∆ = 0.62 %).

**Table 3:** Abdominal fat pad, as percentage of live BW, of broiler breeders from 12 to 60 wk of age with 2 different growth curves (SGC = standard growth curve or EGC = elevated growth curve (+15%)) and 4 diets, differing in energy-to-protein ratio (96, 100, 104, or 108% AME_n_), fed from 0 to 60 wk of age.

		Age (wk)								
Item		12	16	21	24	28	31	36	46	60
Growth curve (n = 12)										
SGC		0.4	0.3^b^	1.1^b^	1.5	1.6^b^	1.5^b^	1.9^b^	1.5	1.7
EGC		0.4	0.6^a^	1.5^a^	1.7	2.3^a^	2.5^a^	2.8^a^	2.9	2.3
SEM		0.0	0.0	0.1	0.1	0.1	0.1	0.1	0.1	0.2
Diet (n = 6)										
96% AME_n_		0.3	0.4	1.0^b^	1.1^b^	1.5^b^	1.5^c^	1.9	2.5	2.4
100% AME_n_		0.4	0.4	1.1^b^	1.4^b^	1.9^ab^	2.0^b^	2.4	2.1	1.6
104% AME_n_		0.4	0.4	1.3^ab^	1.9^a^	2.0^a^	2.1^b^	2.5	2.2	2.0
108% AME_n_		0.5	0.6	1.7^a^	2.0^a^	2.3^a^	2.4^a^	2.7	2.3	2.1
SEM		0.0	0.0	0.1	0.1	0.1	0.1	0.1	0.1	0.2
Treatment (n = 3)										
SGC	96% AME_n_	0.2	0.3	0.9	0.9	1.3	0.9	1.6	1.8	2.3
	100% AME_n_	0.3	0.3	0.9	1.1	1.6	1.5	2.1	1.1	1.3
	104% AME_n_	0.4	0.3	1.2	1.9	1.6	1.4	1.9	1.6	1.9
	108% AME_n_	0.4	0.5	1.4	1.9	1.7	2.2	2.1	1.7	1.5
EGC	96% AME_n_	0.3	0.6	1.2	1.2	1.8	2.0	2.1	3.2	2.5
	100% AME_n_	0.4	0.5	1.3	1.7	2.2	2.4	2.8	3.0	1.9
	104% AME_n_	0.4	0.4	1.4	1.8	2.5	2.7	3.0	2.7	2.2
	108% AME_n_	0.6	0.8	2.0	2.1	2.9	2.7	3.2	2.9	2.7
	SEM	0.1	0.1	0.2	0.2	0.2	0.1	0.3	0.3	0.4
*P*-value^1^										
Growth curve (GC)		0.20	0.005	0.01	0.15	<0.001	<0.001	0.001	<0.001	0.10
Diet (factorial)		0.06	0.07	0.02	0.002	0.03	<0.001	0.10	0.51	0.45
Diet (linear)		0.009	0.06	0.001	<0.001	0.002	<0.001	0.02	0.56	0.74
GC x Diet (factorial)		0.31	0.94	0.69	0.44	0.40	0.06	0.67	0.60	0.72
GC x Diet (linear)		0.76	0.72	0.48	0.55	0.10	0.23	0.23	0.47	0.38

^a-c^LSmeans within a column and factor lacking a common superscript differ (*P ≤* 0.05).

^1^Quadratic contrasts were not significant.

### Egg Production Traits

No interaction between GC and dietary energy-to-protein ratio on sexual maturation was observed (Table 4). The EGC breeders reached sexual maturity earlier (∆ = 4.1 d; *P* < 0.001) and had an earlier production of settable eggs (∆ = 6.5 d; *P* < 0.001) than the SGC breeders. For both GC, a linear increase in dietary energy-to-protein ratio led to a later age at sexual maturity (β = 0.14 d/% AME_n_; *P* = 0.007) and later age at first settable egg (β = 0.39 d/% AME_n_; *P* < 0.001). An interaction between GC and dietary energy-to-protein ratio on peak egg production was observed (*P* = 0.03; Table 4). Peak egg production of breeders slightly increased with an increase in dietary energy-to-protein ratio within the SGC (β = 0.1 %/% AME_n_), but within the EGC, a decrease in peak egg production was observed with an increase in dietary energy-to-protein ratio (β = -0.4 %/% AME_n_).

**Table 4:** Age at sexual maturity, age at first hatching egg, peak egg production, and egg weight during first phase of lay (22–40 wk), second phase of lay (41–60 wk), and total laying phase (22–60 wk) of broiler breeders with 2 different growth curves (SGC = standard growth curve or EGC = elevated growth curve (+15%)) and 4 diets, differing in energy-to-protein ratio (96, 100, 104, or 108% AME_n_), fed from 0 to 60 wk of age.

		Age at sexual maturity^1^ (d)	Age first hatching egg^2^ (d)	Peak egg production^3^ (%)	Egg weight (g)		
Item					24–40 wk	41–60 wk	24–60 wk
Growth curve (n = 12)							
SGC		174.5^a^	177.3^a^	91.4	57.2^b^	67.3^b^	62.6^b^
EGC		170.4^b^	170.8^b^	89.2	59.7^a^	69.4^a^	64.9^a^
SEM		0.3	0.5	0.6	0.1	0.2	0.2
Diet (n = 6)							
96% AME_n_		171.7	172.1^c^	91.0	58.9^a^	68.7	64.2
100% AME_n_		171.9	173.0^bc^	91.3	58.6^ab^	68.3	63.8
104% AME_n_		172.7	174.5^b^	89.7	58.2^b^	68.2	63.6
108% AME_n_		173.3	176.7^a^	89.2	58.2^b^	68.1	63.5
SEM		0.4	0.7	0.8	0.2	0.3	0.2
Treatment (n = 3)							
SGC	96% AME_n_	174.2	175.0	90.1^abc^	57.4	67.1	62.6
	100% AME_n_	173.7	176.2	92.4^a^	57.2	67.2	62.6
	104% AME_n_	174.6	178.4	92.4^a^	57.1	67.4	62.7
	108% AME_n_	175.4	179.8	90.8^ab^	57.1	67.4	62.6
EGC	96% AME_n_	169.2	169.2	91.9^a^	60.2	70.4	65.7
	100% AME_n_	170.2	169.8	90.3^abc^	60.1	69.3	65.1
	104% AME_n_	170.9	170.7	87.1^c^	59.3	69.0	64.5
	108% AME_n_	171.1	173.7	87.7^bc^	59.2	68.8	64.4
SEM		0.6	1.0	1.2	0.3	0.4	0.3
*P*-value^4^							
Growth curve (GC)		<0.001	<0.001	0.02	<0.001	<0.001	<0.001
Diet (factorial)		0.07	0.001	0.27	0.05	0.39	0.19
Diet (linear)		0.007	<0.001	0.08	0.007	0.11	0.03
GC x Diet (factorial)		0.61	0.76	0.05	0.41	0.11	0.16
GC x Diet (linear)		0.65	0.67	0.03	0.12	0.02	0.03

^a-c^LSmeans within a column and factor lacking a common superscript differ (*P* ≤ 0.05).

1 Defined as age at 50% production.

2 Defined as age at egg weight 50 g.

3 Determined as a 3-wk moving average of %/breeder/d.

4 Quadratic contrasts were not significant.

Throughout the laying phase (22–60 wk), egg weight was higher for EGC breeders (∆ = 2.3 g on average; *P* < 0.001) than for SGC breeders (Table 4). The effect of dietary energy-to-protein ratio on egg weight was much smaller, differed between phase of lay and was dependent on GC. In the first phase of lay, a linear increase in dietary energy-to-protein ratio led to a linear decrease in egg weight (β = -0.06 g/% AME_n_; *P* = 0.007), for breeders in both GC. In the second phase of lay, an interaction between GC and diet (linear) was observed (*P* = 0.02). Within EGC breeders, an increase in dietary energy-to-protein ratio led to a linear decrease in egg weight (β = -0.13 g/% AME_n_), whereas within SGC breeders, an increase in dietary energy-to-protein ratio led to a linear increase in egg weight (β = 0.03 g/% AME_n_).

During the first phase of lay, EGC breeders produced a higher total number of eggs per breeder (∆ = 2.5 eggs; *P* = 0.02; Table 5) than SGC breeders. During the second phase of lay, no difference between GC were observed on total number of eggs per breeder produced (Table 5). The EGC breeders produced more unsettable eggs during the first (∆ = 1.6 eggs; *P* < 0.001) and second phase of lay (∆ = 0.6 eggs; *P* = 0.02) than the SGC breeders. Over the whole laying phase (wk 22 to 60), EGC breeders had a higher production of unsettable eggs (∆ = 2.3 eggs; *P* < 0.001) than SGC breeders. The GC effect was observed in almost all categories of unsettable eggs. The EGC breeders produced more double yolked eggs (2.2 vs. 0.9 eggs; *P* < 0.001), dirty eggs (1.5 vs. 0.9 eggs; *P* = 0.002), abnormal shell eggs (0.7 vs. 0.4 eggs; *P* = 0.002), and cracked shell eggs (1.0 vs. 0.7 eggs; *P* = 0.006) than the SGC breeders. No differences between GC were observed in number of small eggs or floor eggs.

**Table 5:** Egg production traits (n/breeder) during first phase of lay (22–40 wk), second phase of lay (41–60 wk), and total laying phase (22–60 wk) of broiler breeders with 2 different growth curves (SGC = standard growth curve or EGC = elevated growth curve (+15%)) and 4 diets, differing in energy-to-protein ratio (96, 100, 104, or 108% AME_n_), fed from 0 to 60 wk of age.

		22–40 wk			41–60 wk			22–60 wk		
Item		Total eggs	Settable eggs^1^	Unsettable eggs^2^	Total eggs	Settable eggs^1^	Unsettable eggs^2^	Total eggs	Settable eggs^1^	Unsettable eggs^2^
Growth curve (n = 12)										
SGC		93.2^b^	86.4	6.9^b^	95.9	94.8	1.2^b^	189.1	181.1	8.0^b^
EGC		95.7^a^	87.2	8.5^a^	97.2	95.4	1.8^a^	192.9	182.6	10.3^a^
SEM		0.6	0.6	0.2	1.8	1.8	0.1	2.2	2.3	0.3
Diet (n = 6)										
96% AME_n_		95.9	89.0^a^	7.0^b^	97.6	96.2	1.4	193.5	185.2	8.3
100% AME_n_		95.6	88.3^ab^	7.3^b^	95.5	93.9	1.6	191.1	182.3	8.9
104% AME_n_		94.1	86.0^bc^	8.1^ab^	96.7	95.2	1.5	190.7	181.2	9.5
108% AME_n_		92.3	83.8^c^	8.5^a^	96.3	95.0	1.4	188.6	178.7	9.8
SEM		0.9	0.9	0.3	2.6	2.7	0.2	3.4	3.4	0.4
Treatment (n = 3)										
SGC	96% AME_n_	93.1	86.8	6.3	96.5	95.3	1.1	189.6	182.2	7.4
	100% AME_n_	94.0	87.2	6.8	93.5	92.4	1.1	187.5	179.6	7.9
	104% AME_n_	94.4	87.0	7.4	98.7	97.5	1.2	193.0	184.5	8.6
	108% AME_n_	91.4	84.4	7.0	95.0	93.8	1.1	186.3	178.2	8.1
EGC	96% AME_n_	98.8	91.1	7.7	98.7	97.1	1.6	197.4	188.2	9.2
	100% AME_n_	97.2	89.4	7.8	97.6	95.5	2.0	194.8	184.9	9.8
	104% AME_n_	93.8	85.0	8.7	94.7	92.9	1.8	188.4	177.9	10.5
	108% AME_n_	93.1	83.2	9.9	97.7	96.1	1.6	190.8	179.3	11.6
	SEM	1.3	1.2	0.5	3.8	3.9	0.3	4.9	4.9	0.6
*P*-value^3^										
Growth curve (GC)		0.02	0.36	<0.001	0.66	0.82	0.02	0.32	0.69	<0.001
Diet (factorial)		0.06	0.004	0.05	0.96	0.95	0.87	0.82	0.65	0.15
Diet (linear)		0.006	<0.001	0.004	0.83	0.84	0.99	0.35	0.21	0.02
GC x Diet (factorial)		0.16	0.08	0.27	0.73	0.75	0.88	0.60	0.59	0.56
GC x Diet (linear)		0.08	0.02	0.13	0.79	0.80	0.96	0.49	0.40	0.23

^a-c^LSmeans within a column and factor lacking a common superscript differ (*P* ≤ 0.05).

1 Settable egg = clean egg (≥50 g).

2 Unsettable egg = small (<50 g), double yolk, abnormal shell, dirty or floor egg.

3 Quadratic contrasts were not significant.

The effect of dietary energy-to-protein ratio on total and unsettable egg production differed per phase of lay (Table 5). During the first phase of lay, an increase in dietary energy-to-protein ratio linearly decreased total number of eggs (β = -0.31 eggs/% AME_n_; *P* = 0.006) and linearly increased number of unsettable eggs (β = 0.13 eggs/% AME_n_; *P* = 0.004; Table 5). This was mainly due to a linear increase in double yolked eggs (β = 0.05 double yolked eggs/% AME_n_; *P* = 0.01) and small eggs (β = 0.07 small eggs/% AME_n_; *P* = 0.03). In the second phase of lay, no significant effects of dietary energy-to-protein ratio were observed on total and unsettable egg production.

A linear interaction between GC and dietary energy-to-protein ratio was observed on settable egg production (Table 5). An increase in dietary energy-to-protein ratio linearly decreased settable egg production (*P* < 0.001) in both GC, but the effect on settable egg production was more profound within EGC breeders (β = -0.70 eggs/% AME_n_) than within SGC breeders (β = -0.19 eggs/% AME_n_; GC x Diet (linear), *P* = 0.02). In the second phase of lay, no significant effects of dietary energy-to-protein ratio were observed on settable egg production.

### Feather Development

A linear interaction between GC and dietary energy-to-protein ratio was observed on average feather cover score (Table 6; GC x Diet (linear), *P* = 0.04). Within SGC breeders, a linear increase in dietary energy-to-protein ratio led to a linear increase in feather cover score (β = 0.02 points/% AME_n_), whereas this linear effect was not observed within EGC breeders (β = 0.00 points/% AME_n_). Feather cover score increased with breeder age (*P* < 0.001). Feather weight did not differ between treatments (Table 6).

**Table 6:** Average feather cover score and feather weight from 5 to 60 wk of age, of broiler breeders with 2 different growth curves (SGC = standard growth curve or EGC = elevated growth curve (+15%)) and 4 diets, differing in energy-to-protein ratio (96, 100, 104, or 108% AME_n_), fed from 0 to 60 wk of age.

		Feather cover score^1^					Feather weight
Item		Back	Tail	Thigh	Wing	Average	(% of BW)
Growth curve (n = 12)							
SGC		1.18	1.14	1.50^b^	1.42	1.31	3.8
EGC		1.25	1.16	1.61^a^	1.45	1.37	3.7
SEM		0.1	0.1	0.1	0.1	0.1	0.1
Diet (n = 6)							
96% AME_n_		1.19	1.11	1.56^ab^	1.38	1.31^b^	3.8
100% AME_n_		1.18	1.11	1.51^b^	1.43	1.31^b^	3.7
104% AME_n_		1.22	1.14	1.51^b^	1.41	1.30^b^	3.7
108% AME_n_		1.28	1.24	1.65^a^	1.51	1.42^a^	3.8
SEM		0.1	0.1	0.1	0.1	0.1	0.1
Treatment (n = 3)							
SGC	96% AME_n_	1.06	1.05	1.44	1.28	1.21	3.8
	100% AME_n_	1.23	1.11	1.46	1.45	1.31	3.7
	104% AME_n_	1.18	1.13	1.46	1.41	1.20	3.8
	108% AME_n_	1.25	1.27	1.64	1.53	1.42	3.8
EGC	96% AME_n_	1.31	1.16	1.69	1.49	1.41	3.8
	100% AME_n_	1.13	1.10	1.55	1.40	1.30	3.7
	104% AME_n_	1.26	1.15	1.55	1.42	1.35	3.6
	108% AME_n_	1.31	1.21	1.66	1.49	1.42	3.7
	SEM	0.1	0.1	0.1	0.1	0.1	0.1
*P*-value^2^							
Growth curve (GC)		0.43	0.91	0.003	0.43	0.33	0.51
Diet (factorial)		0.26	0.06	0.03	0.23	0.002	0.71
Diet (linear)		0.08	0.020	0.11	0.07	<0.001	0.62
Diet (quadratic)		0.41	0.20	0.007	0.54	0.12	0.32
Age		<0.001	<0.001	<0.001	<0.001	<0.001	<0.001
GC x Diet (factorial)		0.08	0.37	0.18	0.14	0.07	0.81
GC x Diet (linear)		0.47	0.10	0.04	0.08	0.04	0.96
GC x Age		0.53	0.82	<0.001	0.26	0.11	0.19

ab LSmeans within a column and factor lacking a common superscript differ (*P* ≤ 0.05).

1 Feather cover score ranges from 0 (intact feathers) to 5 (completely denuded area). Each value is a mean of the replicates determined at a 5-wk interval during rearing (0–21 wk) and a 10-wk interval during production (21–60 wk).

2 Interactions between GC and Diet (quadratic), between Diet and Age and between Diet, Growth curve, and Age were not significant.

## DISCUSSION

The objective of this study was to evaluate effects of GC and dietary energy-to-protein ratio during rearing and production on productive performance of broiler breeder hens. Interactions will be discussed within the section of dietary energy-to-protein ratio.

### Growth Curve

In the current study, pullets and breeders required on average a 15.1% higher feed allocation to obtain a 15% higher BW. A higher feed allocation to achieve a higher BW is in line with other studies (Renema et al., 2001b; Gous and Cherry, 2004; Ekmay et al., 2012; Van Emous et al., 2013; De Los Mozos et al., 2017; Van der Klein et al., 2018). In the current study, the extra feed allocation and thus higher BW resulted in a higher abdominal fat pad. This was according to expectations and in line with other studies (Renema et al., 2001b; Robinson et al., 2007; Van Emous et al., 2013; Van der Klein et al., 2018; Salas et al., 2019), as body composition is related to feed allocation (Robinson et al., 2007) and diet composition (Spratt and Leeson, 1987).

Sexual maturation occurred 4.1 d earlier in breeders of the EGC than in breeders of the SGC. Other studies reported similar findings, with a 2.8 d (24% heavier) to 15 d (74% heavier) earlier sexual maturity for heavier breeders (Renema et al., 2001b, 2007a; Gous and Cherry, 2004; Sun and Coon, 2005; Ekmay et al., 2012; Van der Klein et al., 2018). Sexual maturation depends on physiological cues, such as photorefractory and photosensitivity (Gous and Cherry, 2004; Van der Klein et al., 2018; Van Emous et al., 2018), but metabolic cues are suggested to play a role as well (Bédécarrats et al., 2016; Hanlon et al., 2020; Van der Klein et al., 2020). It has been suggested that broiler breeders require a certain protein threshold for sexual maturation (Sun et al., 2006; Eitan et al., 2014; Salas et al., 2019), whereas others emphasize the presence of a fat threshold for sexual maturation (Van der Klein et al., 2018; Zuidhof, 2018; Hadinia et al., 2020). In the current study, a 1.6% higher abdominal fat pad was observed at 21 wk of age for EGC breeders compared to SGC breeders, indicating an approximately 84 g higher body fat mass (Zuidhof, 2018). As BW difference between the GC was 406 g at 21 wk of age, the remaining difference in BW can probably be attributed to an increased lean and ash mass. A higher BW thus appears to be the result of both a higher body protein and a higher body fat mass and based on these, it is unclear whether a higher protein or higher fat mass is the main driver for sexual maturation in breeders.

Besides an earlier age at sexual maturity, EGC breeders laid on average 2.3 g heavier eggs than SGC breeders. Other studies observed no effect of a 13 to 20% higher BW at 20 wk of age on egg weight (Gous and Cherry, 2004; Renema et al., 2007a; Ekmay et al., 2012; Van Emous et al., 2013; Van der Klein et al., 2018; Salas et al., 2019) or only a 0.9 g (Sun and Coon, 2005) to 1.9 g (Renema et al., 2001a) increase in egg weight. Some of these studies converged different GC up to 20 wk of age to a similar BW during production (Sun and Coon, 2005; Renema et al., 2007a; Van Emous et al., 2013), whereas other studies fixed either GC, and thus BW gain (Van der Klein et al., 2018), or feed allowance (Gous and Cherry, 2004; Ekmay et al., 2012; Salas et al., 2019) during the laying phase, irrespective of BW at 20 wk of age. The amount of feed available for growth and egg production depends on the amount of feed that is used for maintenance. The latter one being mainly dependent on BW (Caldas et al., 2018; Hadinia et al., 2018) and to a lesser extent on body composition (Gous, 2015). A fixed BW, GC, or feed allowance during the laying phase probably reduced the amount of feed available for growth and egg production for heavier breeders compared to lighter breeders. In this way, lighter breeders may benefit from this, as they have more nutrients available for egg production and consequently produce similar egg weights as heavier breeders (Gous and Cherry, 2004; Sun and Coon, 2005; Renema et al., 2007a; Ekmay et al., 2012; Van Emous et al., 2013; Van der Klein et al., 2018; Salas et al., 2019). In the current study and that of Renema et al. (2001a), a relative difference in GC throughout production was maintained by a higher feed allowance for the EGC breeders compared to SGC breeders. With this feeding strategy, EGC breeders receive the same relative amount of nutrients for maintenance, growth, and egg production as the SGC breeders. The results thus imply that a higher GC leads to heavier eggs, only when a difference in GC and thus feed allowance is maintained during production. A higher egg weight, in turn, might be beneficial for day-old chick quality (Ulmer-Franco et al., 2010; Nangsuay et al., 2011). Further research should investigate the impact of GC on offspring quality and performance.

In the first phase of lay, EGC breeders had a higher total egg production compared to SGC breeders. This was also observed by Ekmay et al. (2012) and can probably be explained by a longer laying phase, as a result of an earlier start of production. However, number of settable eggs did not differ between both GC, as the EGC breeders had a higher number of unsettable eggs. Similar findings were observed in other studies, where heavier breeders had a higher number of unsettable eggs (Renema et al., 2001a; Gous and Cherry, 2004; Sun and Coon, 2005; Sun et al., 2006). Van der Klein et al. (2020) extensively reviewed mechanisms associated with reproductive dysregulation. They suggested that metabolic status and feed allowance affect endocrine (dys)regulation of follicle selection and maturation. However, mechanisms are complex and not yet fully elucidated (Van der Klein et al., 2020).

Over the whole laying phase, no difference in total or settable number of eggs was observed between both GC, which is in line with others (Renema et al., 2001a; Gous and Cherry, 2004; Sun and Coon, 2005; Salas et al., 2019). These results implicate that a 15% higher GC is neither beneficial nor detrimental for total number of settable eggs. However, it is known that ad libitum feeding of breeders is detrimental for total number of eggs produced (Robinson et al., 1991; Bruggeman et al., 1999; Heck et al., 2004; Sun et al., 2006). This suggests there is an upper limit, which might be related to either BW, body protein, or body fat mass, until which egg production is unaffected. When this limit is exceeded, egg production may drop.

### Dietary Energy-to-Protein Ratio

Pullets and breeders fed a diet with a higher dietary energy-to-protein ratio required less feed to achieve a similar BW. This is in line with previous studies, where a higher dietary energy-to-protein ratio was achieved by a higher energy content (Moraes et al., 2014; Van Emous et al., 2015). However, in studies where a higher dietary energy-to-protein ratio was achieved by a lower protein content an opposite relation was observed. In that case, a higher dietary energy-to-protein ratio required a higher feed allocation to achieve a similar BW (Van Emous et al., 2013; Moraes et al., 2014; Lesuisse et al., 2017, 2018). These results indicate that dietary energy-to-protein ratio per se and feed allocation are not correlated to achieve a certain target BW. However, when diets have either a higher energy or a higher protein content, consequently changing the ratio, a lower feed allocation is required to achieve a similar BW. These studies indicate that absolute intake of energy or protein determines growth in breeders, rather than the ratio between them.

The current study indicated that an absolute intake of energy determined growth in pullets and not protein intake. During the rearing phase, it was observed that pair-gaining of pullets required a similar energy intake between the different diets, while protein intake differed. Moraes et al. (2014) also observed a similar energy intake between diets different in energy-to-protein ratio, to achieve pair-gaining during the rearing phase. Feed restriction of pullets might play a role in this. In broilers, it is observed that, when feed restriction is applied, energy intake is limiting for growth of broilers (Boekholt et al., 1994; Leeson et al., 1996). Pullets cannot compensate for an energy limitation by increasing their feed intake, as is observed in ad libitum fed broilers (Leeson et al., 1996; Yang et al., 2009). Conversion of dietary protein and energy into body protein and body fat requires a minimum energy intake (Boekholt et al., 1994). Energy intake might therefore limit growth in pullets.

After peak production, SD within the SGC was lower with an increasing energy-to-protein ratio, whereas SD within the EGC, was higher with an increasing energy-to-protein ratio. Management plays a major role in BW uniformity (Aviagen, 2018), where a similar feed intake among breeders within a flock is key in aiming for a high BW uniformity (Zuidhof, 2018). A larger amount of feed gives less dominant breeders an opportunity to compete for feed, which might lead to a higher BW uniformity (De Beer and Coon, 2009). This is in line with observations within the EGC, as a higher feed allocation corresponded with a lower SD and thus a higher BW uniformity during production. However, within the SGC, breeders fed the lowest amount of feed had the lowest SD and thus a better BW uniformity than breeders with a higher amount of feed. A more severe feed restriction leads to a higher eating rate (De Los Mozos et al., 2017). It can be speculated that feed allocation was so restricted for breeders on the 104% and 108% AME_n_ diet, within the SGC, that the daily feed portion was consumed in 1 feeding bout in a short time, leading to a uniform feed intake for all breeders within a pen. For the 96% and 100% AME_n_, within the SGC, the daily feed portion might not have been consumed in 1 feeding bout, due to a limitation in physical eating capacity. In that case, breeders with a higher digestive capacity or more dominant breeders might have had the opportunity to consume a second feeding portion or a larger portion, leading to a decline in BW uniformity.

An increase in dietary energy-to-protein ratio led to a linear increase in abdominal fat pad and thus a higher body fat mass (Zuidhof, 2018). This is in line with other studies (Van Emous et al., 2013, 2015; Lesuisse et al., 2017, 2018; Salas et al., 2019). Growing animals always have a basic protein retention (Boekholt et al., 1994; Boekholt and Schreurs, 1997) and in case a surplus of energy is supplied, this is mostly retained as fat (Boekholt et al., 1994; Leeson et al., 1996). A higher dietary energy-to-protein ratio supplies pullets and breeders with a surplus of energy, which is deposited as fat. Interestingly, all diets had a decreased abdominal fat pad percentage between 36 and 60 wk of age, with exception of 96% AME_n_ diet. A reduction in fat mass after peak production was also observed by Salas et al. (2019). Fat in egg yolk is highly depended on body fat mobilization during late production (Salas et al., 2017), suggesting that body fat mobilization after 36 wk of age supports egg production. Breeders fed the 96% AME_n_ diet had a further increase in abdominal fat pad between 36 and 46 wk of age, prior to a decrease in abdominal fat pad between 46 and 60 wk of age. These breeders required a relative high feed allocation, compared to the other diets, between 36 and 46 wk of age to achieve pair-gaining. This higher feed allocation and nutrient intake probably resulted in deposition of fat in the body. These results might indicate that these breeders were inefficient with their nutrients in this period, potentially due to an imbalance between energy and protein in the diet.

A change in body composition might have influenced sexual maturation (Bédécarrats et al., 2016; Hanlon et al., 2020; Van der Klein et al., 2020). Breeders fed a higher dietary energy-to-protein ratio had a later age at sexual maturity. However, others did not observe an effect of dietary energy-to-protein ratio on sexual maturation (Joseph et al., 2002; Van Emous et al., 2013, 2015, 2018; England et al., 2014; Lesuisse et al., 2017, 2018; Salas et al., 2019). Contradictory to the current study, all these studies altered dietary energy-to-protein ratio by adjusting protein content of the diet, with exemption of the study of Salas et al. (2019). Additionally, the current study design allowed linear analysis of dietary energy-to-protein ratio, whereas all other studies had a factorial design. Linear analysis of our data showed a significant effect of dietary energy-to-protein ratio on sexual maturation, whereas the factorial analysis did not show this. Salas et al. (2019) only started their dietary treatments at 20 wk of age, which indicates that energy content during the rearing phase might play a role in sexual maturation. Broiler breeders require a protein or fat threshold for sexual maturation (Zuidhof, 2018; Salas et al., 2019; Hadinia et al., 2020). Breeders with a higher abdominal fat pad percentage, and thus a higher body fat mass (Zuidhof, 2018), at 21 wk of age had a later age at sexual maturation compared to breeders with a lower fat pad percentage, at a similar BW. These results suggest that a fat threshold did not play an important role in sexual maturation. Future studies should consider analysis of body protein and body fat content at different ages during rearing to determine their importance for sexual maturation.

An increase in energy-to-protein ratio led to a decrease in average egg weight. On average a 0.7 g lower egg weight was observed for the 108% AME_n_ diet than for the 96% AME_n_ diet. This is comparable with Van Emous et al. (2015), who observed a 0.4 g lower egg weight, with 7.1% higher energy content in the diet. Spratt and Leeson (1987) and Sun and Coon (2005) observed a 1.8 g and 0.85 g higher egg weight with 38% and 5.4% higher energy content in the diet, respectively. However, those studies were based on pair-feeding instead of pair-gaining, meaning the higher energy content group also had a higher BW and consequently a higher egg weight. Other studies did not observe an effect of 8.2% (Moraes, 2013) to 45% (Salas et al., 2019) higher energy content on egg weight. The latter one might be explained by a similar protein intake, as protein content and intake seems to have a more profound effect on egg weight than energy intake. Dietary protein is an important source of egg protein for egg formation (Ekmay et al., 2014) and consequently a reduced protein intake during lay might influence egg weight. A reduction in protein content in the diet (12.5 % to 25%) resulted in a 0.7 g to 4.7 g lower egg weight (Spratt and Leeson, 1987; Joseph et al., 2000; England et al., 2014; Lesuisse et al., 2017, 2018). These effects were only observed when fed during the laying phase, as a lower crude protein content during the rearing phase alone did not affect egg weight in the laying phase (Moraes, 2013; Van Emous et al., 2013, 2015). Further research should investigate whether or not the ratio between egg yolk and albumen is influenced as well by changing the dietary energy-to-protein ratio, as this might eventually affect chick quality (Finkler et al., 1998; Willems et al., 2015).

An earlier start of production and an earlier age at first settable egg, due to a higher egg weight, almost entirely explains the higher settable egg production during the first phase of lay for breeders fed a lower dietary energy-to-protein ratio. This is in line with observations from other studies (Joseph et al., 2000; Lesuisse et al., 2017, 2018), who reduced crude protein content by 25% in the diet. However, studies that reduced crude protein or energy content by 5% to 20% did not observe an effect on egg production during the first phase of lay (Joseph et al., 2002; Sun and Coon, 2005; Van Emous et al., 2013, 2015, 2018; England et al., 2014). Maybe more important, these studies only fed a diet lower in protein during either the rearing (Van Emous et al., 2013, 2015) or the laying phase (Joseph et al., 2002; Sun and Coon, 2005; England et al., 2014; Van Emous et al., 2018). This suggests that a lower dietary energy-to-protein ratio might be beneficial for egg production during the first phase of lay, but only when diets are fed both during rearing and first phase of lay.

In line with Sun and Coon (2005) and Salas et al. (2019), in the second phase of lay and over the total laying phase, no differences were observed in total and settable egg production. Contradictory, in the study of Van Emous et al. (2015), a higher dietary energy-to-protein ratio during rearing improved persistency between wk 45 and 60 of age. They speculated that a higher fat mass and a lower lean mass at 20 wk of age was beneficial for laying persistency. Body fat mass needs relative low maintenance (Gous, 2015), which might increases amount of energy available for egg production for fatter breeders than for leaner breeders. The current study did not observe a difference between diets in abdominal fat pad percentage at 46 and 60 wk of age, which might explain the lack of differences in egg production during the second phase of lay.

## CONCLUSIONS

It can be concluded that a higher GC of breeders during rearing and production led to an earlier age at sexual maturation and a higher egg weight, but did not affect total number of settable eggs produced. Feeding breeders a lower dietary energy-to-protein ratio, while maintaining a similar GC, led to a lower abdominal fat pad and an earlier age at sexual maturation. In the first phase of lay, feeding a lower dietary energy-to-protein ratio led to a higher egg weight and a higher number of settable eggs produced. This dietary effect was more profound when breeders were on a higher GC. In the second phase of lay, feeding a lower dietary energy-to-protein ratio led to a higher egg weight when breeders were on a higher GC, but not when breeders were on a standard GC. Dietary energy-to-protein ratio did not affect number of settable eggs produced in the second phase of lay. A higher body fat mass, within a similar BW, thus does not have beneficial effects on productive performance. We suggest further research to investigate the impact of GC and dietary energy-to-protein ratio on egg composition and offspring performance.

## ACKNOWLEDGMENTS

The authors wish to thank De Heus Animal Nutrition B. V. for funding this project, Aviagen-EPI for providing the day-old broiler breeder pullets, and the animal caretakers from V. O. F. De Horizon, MSc students Emma Beijer, Roseanne Minderhoud, Stephanie Ten Kate, and Gydo Broskij for their assistance.

## Disclosures

Authors J. Heijmans, and M. Duijster are employed by company De Heus Animal Nutrition B.V. All authors declare that the research was conducted in absence of any commercial or financial relationships that could be construed as a potential conflict of interest.
